# Airway Management During Laparoscopic Surgery in a Patient With Saber Sheath Trachea: A Case Report

**DOI:** 10.7759/cureus.102611

**Published:** 2026-01-30

**Authors:** Hibiki Saito, Koichi Maruyama, Asako Iwashita, Sayaka Saito, Tomio Andoh

**Affiliations:** 1 Anesthesiology, Mizonokuchi Hospital, Teikyo University School of Medicine, Kawasaki, JPN

**Keywords:** 3-dimensional computed tomography, airway management, supraglottic airway device, tracheal narrowing, tracheomalacia

## Abstract

Saber-sheath trachea is a U-shaped diffuse narrowing of the thoracic trachea, specifically observed in patients with chronic obstructive pulmonary disease. The airway management of patients with saber-sheath trachea can be challenging because it possesses characteristics both of tracheal stenosis and tracheomalacia. A 78-year-old man was diagnosed with gastric cancer and underwent robot-assisted laparoscopic total gastrectomy with jejunal interposition reconstruction. We provided general anesthesia combined with thoracic epidural anesthesia for this case. For intraoperative airway management, we chose the ProSeal laryngeal mask airway (PLMA) (Senko Medical Instrument Mfg. Co., Ltd., Tokyo, Japan) to avoid potential problems associated with tracheal intubation. We did not experience any surgical difficulties during intraoperative mechanical ventilation or tracheal collapse during the course of the recovery of spontaneous ventilation and emergence from anesthesia. We believe that the use of second-generation supraglottic airway devices with esophageal drainage tubes may be considered as an alternative option for airway management in similar cases involving saber-sheath trachea.

## Introduction

The saber-sheath trachea deformity presents diffuse tracheal narrowing of the thoracic cavity, characterized by shortening of the coronal diameter and concomitant widening of the sagittal diameter [1]. The ratio of the sagittal to the coronal diameter in the intrathoracic segment (tracheal index) exceeds 2:1. Otherwise, the extra-thoracic trachea is normal [2]. This entity is a pathognomonic finding in patients with chronic obstructive pulmonary disease. It is believed that these patients suffer from mechanical injury caused by recurrent coughing, which leads to cartilaginous degeneration and repair. In the process, an abnormal U-shaped tracheal structure is formed due to prolonged elevation of intrathoracic pressure [3, 4]. The walls of the saber-sheath trachea are thick and frequently ossified, making collapse unlikely [2]. However, the deformed trachea also exhibits characteristics of tracheomalacia [5, 6]. In these patients, inward bowing or displacement of the lateral tracheal walls can be observed on computed tomography (CT) scans, which is exacerbated by forced expiration [5]. Due to these complex pathological conditions, the airway management in patients with saber-sheath trachea can be potentially challenging.

We report the case of a patient with a saber-sheath trachea who underwent laparoscopic gastrectomy under general anesthesia using the ProSeal laryngeal mask airway (PLMA) (Senko Medical Instrument Mfg. Co., Ltd., Tokyo, Japan).

## Case presentation

**Figure 1:**
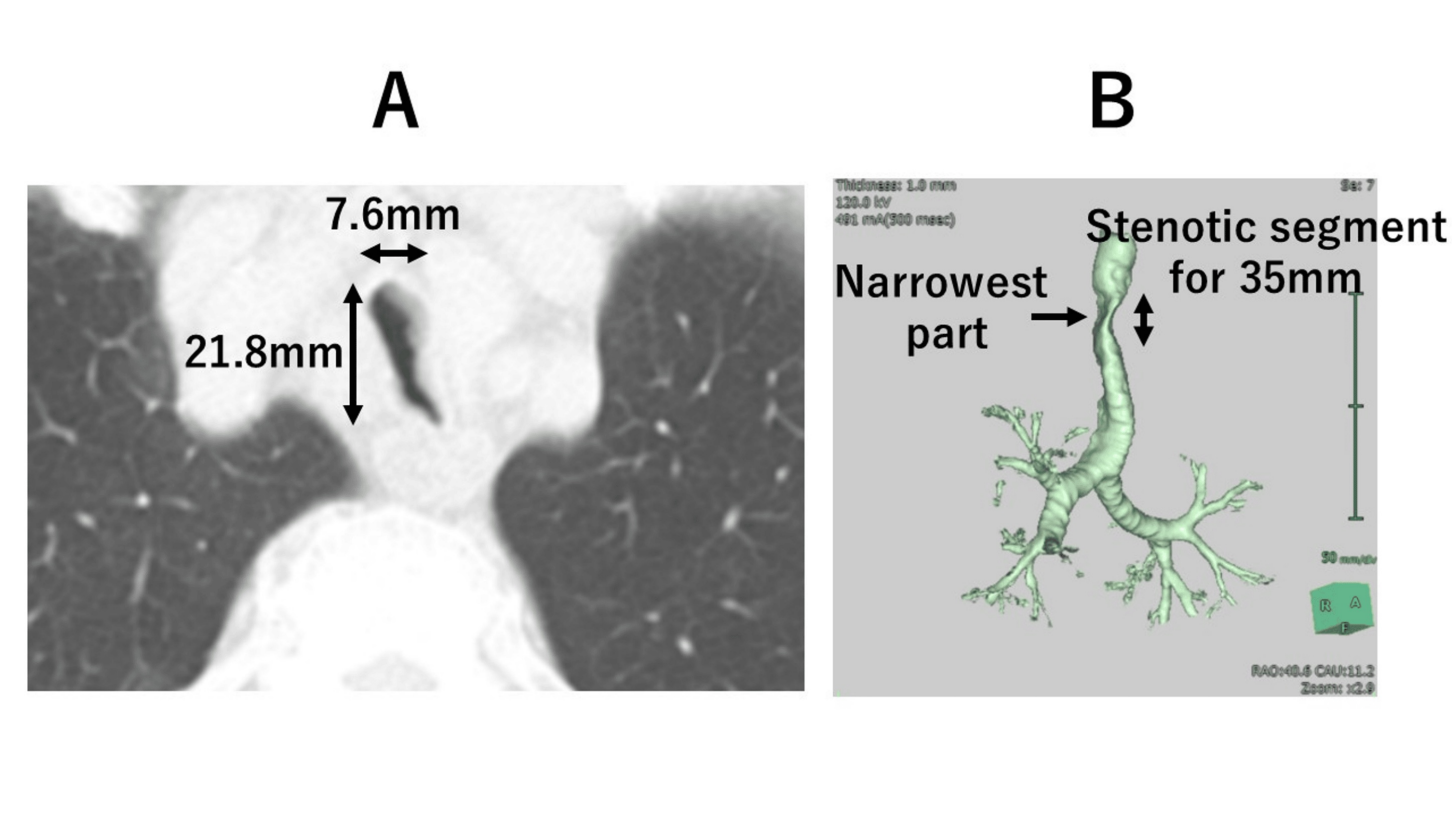
CT images of the trachea A: Preoperative axial CT image showing the narrowest part of the coronal diameter measured approximately 7.6 mm with the sagittal diameter of 21.8 mm. B: Right anterolateral view of the three-dimensional reconstructed trachea showing a markedly stenotic segment with a length of 35 mm.

A 78-year-old man (height, 157.2 cm; weight, 64.1 kg) with gastric cancer was scheduled for robot-assisted laparoscopic total gastrectomy with jejunal interposition reconstruction. Preoperative pulmonary function tests revealed mild obstructive respiratory dysfunction, with a ratio of forced expiratory volume in 1 second to forced vital capacity of 67% and a percent vital capacity of 128%. The patient had a history of heavy smoking (15 cigarettes per day), though there were no respiratory symptoms. Chest CT suggested no apparent imaging findings of pulmonary emphysema but revealed significant intra-thoracic tracheal U-shaped narrowing; the coronal diameter was much smaller than the sagittal diameter. The morphological changes began 30 mm distal to the glottis and extended for 35 mm. The narrowest part of the coronal diameter measured approximately 7.6 mm, while the sagittal diameter was 21.8 mm at 40 mm distal to the glottis and 65 mm proximal to the carina (Figure 1).

These findings were consistent with moderate-to-severe intrathoracic tracheal narrowing with a preserved sagittal diameter, characteristic of saber-sheath trachea.

In the operating room, an electrocardiograph, pulse oximeter, and noninvasive blood pressure monitor were utilized. After the insertion of the thoracic epidural catheter at T10/11, general anesthesia was induced with propofol, remifentanil, and rocuronium. A size 5 PLMA was inserted without difficulty after the significant narrowing of the trachea was confirmed using fiberoptic bronchoscopy. Then, arterial blood pressure monitoring was added, and a gastric tube was inserted through the PLMA drainage tube port. Anesthesia was maintained with an epidural block, sevoflurane, propofol, remifentanil, and continuous rocuronium administration. The PLMA required a fine adjustment of position and a cuff pressure of approximately 40 cmH2O during the operation. However, the patient’s lung was ventilated adequately throughout the procedure (tidal volume, 500 ml; respiratory rate, 12 breaths/min; positive-end expiratory pressure, 5 cmH2O; and peak pressure was almost <20 cmH2O). These values were appropriately adjusted intraoperatively to maintain the end-tidal carbon dioxide at 35-40 mmHg. No surgical difficulties associated with PLMA airway management (e.g., gastric distention) were reported by the surgeon. After finishing the operation, the patient resumed spontaneous ventilation, and the PLMA was removed uneventfully after adequate reversal of the muscle relaxant with sugammadex. The duration of surgery and anesthesia was 8 h 37 min and 9 h 46 min, respectively. Although the epidural catheter was removed accidentally after use for patient-controlled analgesia for approximately 24 h after surgery, the postoperative course was uneventful without any respiratory complications. The patient was discharged from the hospital eight days after surgery.

## Discussion

Airway management with a tracheal tube in patients with saber‑sheath trachea may present significant challenges, especially if the condition is not diagnosed preoperatively. Several case reports have described difficulties in airway management in saber-sheath trachea, including difficulty in ventilating the lungs despite a good laryngoscopic view and visual confirmation of tracheal intubation [7], obstruction of the right-sided opening of the double lumen tube by the narrowed tracheal wall in the left lateral decubitus position [8], and difficulty in achieving an adequate seal with the tracheal tube cuff [9]. A case of negative pressure pulmonary edema was also reported. In that patient, pulmonary edema suddenly developed after resuming spontaneous ventilation on emergence from anesthesia. A subsequent CT scan demonstrated partial collapse of the saber-sheath trachea, while the cardiac function was preserved. The authors mentioned that calcification, stiffening, followed by microfracture and destruction of the normally elastic tracheal rings, or deposition of abnormal collagen, may have contributed to the development of tracheomalacia in saber-sheath trachea [6].

To minimize airway complications associated with saber-sheath trachea, avoidance of tracheal intubation would be ideal, if possible (e.g., by maximizing the use of peripheral nerve blocks or neuraxial blocks) [10].

In this patient, intraoperative mechanical ventilation with a muscle relaxant was required to accomplish the procedure. The coronal diameter at the narrowest point was 7.6 mm. Therefore, we proactively chose this supraglottic airway device (SAD) primarily due to concerns about edema at the stenotic site and the potential for worsening airway stenosis postoperatively, given the prolonged direct rigid compression by the tracheal tube and the inflated cuff.

When SADs are used in laparoscopic surgeries, there are concerns about the risk of aspiration, impaired ventilation, and the risk of gastric regurgitation due to increased intra-abdominal pressure [11]. However, recent studies have demonstrated the successful management of elective laparoscopic surgeries using second-generation SADs with esophageal drainage tubes, without an increased risk of aspiration or gastric distension [12-14]. In this operation, the patient was kept in the reverse Trendelenburg position for most of the procedure, and the airway was managed by an experienced anesthetist. These conditions may have contributed to mitigating the risks associated with SADs.

The use of the PLMA offers additional advantages over tracheal intubation for these patients, especially during emergence from anesthesia. First, tracheal tubes easily induce cough reflex due to direct stimulation of the tracheal mucosa during emergence from anesthesia. Consequent forced expiration followed by bucking can exacerbate the inward bowing of the lateral tracheal walls [5], potentially causing critical tracheal obstruction. In contrast, SADs promote a smooth awakening from anesthesia without physiological tracheal stimulation. Therefore, the PLMA seems to be effective in avoiding tracheal collapse during emergence from anesthesia. Second, the PLMA may be useful in maintaining the glottic function. Previously, a case of negative -pressure pulmonary edema developing during emergence from anesthesia was reported [6]. The authors suggested that prevention of glottal closure by the tracheal tube could lead to loss of continuous positive airway pressure before extubation, resulting in tracheal collapse and subsequent negative-pressure pulmonary edema. This speculation supported the use of the PLMA for airway management in such cases, as it would preserve stenting pressure on the trachea by glottal closure during the expiratory phase when spontaneous breathing resumes during emergence from anesthesia.

## Conclusions

In summary, the airway of a patient with saber-sheath trachea was successfully managed with the PLMA, without ventilatory difficulty, procedural burden, or intraoperative tracheal collapse. No postoperative pulmonary complications were observed. We believe that the use of second-generation SADs with esophageal drainage tubes may be considered as an alternative option for airway management in similar cases involving saber-sheath trachea, though further experience and studies are needed.
